# COVID-19 wastewater surveillance implemented in the Tokyo 2020 Olympic and Paralympic Village

**DOI:** 10.1093/jtm/taac004

**Published:** 2022-02-03

**Authors:** Masaaki Kitajima, Michio Murakami, Ryo Iwamoto, Hiroyuki Katayama, Seiya Imoto

**Affiliations:** Division of Environmental Engineering, Faculty of Engineering, Hokkaido University, Hokkaido, Japan; Center for Infectious Disease Education and Research, Osaka University, Osaka, Japan; Shionogi & Co. Ltd., Osaka, Japan; Department of Urban Engineering, Graduate School of Engineering, The University of Tokyo, Tokyo, Japan; Human Genome Center, The Institute of Medical Science, The University of Tokyo, Tokyo, Japan

## Abstract

Wastewater-based epidemiology was implemented in the Olympic and Paralympic Village to better understand COVID-19 incidence. SARS-CoV-2 RNA was detected in a number of wastewater samples even when no positive individual was identified in the corresponding areas. Wastewater-based epidemiology can be a useful tool to control infections at mass gatherings.

## Text

Wastewater-based epidemiology (WBE), which has attracted attention as a COVID-19 surveillance tool,^1^ was implemented in the Tokyo 2020 Olympic and Paralympic Village to better understand COVID-19 incidence in the village.^2^ Between 14 July and 8 September 2021, 690 wastewater samples—361 and 329 samples collected via passive and grab sampling, respectively—were collected from manholes in the village. We collected wastewater samples, in addition to clinical data (i.e. confirmed positive cases), from seven distinct areas comprising the entire residential buildings. The wastewater samples were examined for the presence and concentration of severe acute respiratory syndrome coronavirus 2 (SARS-CoV-2) RNA using a highly sensitive reverse transcription (RT)-quantitative polymerase chain reaction (qPCR)-based detection method. Briefly, RNA was extracted from the wastewater samples using a kit, and one-step RT-preamplification followed by qPCR was performed using the CDC N1 (2019-nCoV_N1) primers and probe (https://www.cdc.gov/coronavirus/2019-ncov/lab/rt-pcr-panel-primer-probes.html). Negative controls for each process (i.e. RNA extraction, RT-preamplification and qPCR) were always included and no positive signal was observed from any of the negative controls. We tested for SARS-CoV-2 RNA in wastewater and reported data daily to the Tokyo Organising Committee of the Olympic and Paralympic Games. The reported data were used as one of the indicators reflecting COVID-19 incidence to support judgement of the need for enhanced infection prevention measures.

SARS-CoV-2 RNA was detected in a total of 233 (33.8%) wastewater samples consisting of 151 (41.8%) and 82 (24.9%) samples from passive and grab sampling, respectively, even in the areas where no positive case was identified via mandatory daily clinical testing among residents,^3^ which is probably because our method is so sensitive that viral RNA excreted from non-infectious post-quarantine patients and asymptomatic carriers with low viral shedding was detected in the wastewater. In fact, it has been pointed out that asymptomatic carriers with lower viral load would test negative by the Lumipulse® SARS-CoV-2 antigen saliva test employed as the initial screening tool in the village, although it has shown to reliably identify most of those with higher viral load.^4^ When SARS-CoV-2 RNA was not detected via passive sampling of wastewater in a given area for three consecutive days, clinical tests rarely (only 6.7%) identified positive cases in that area. Genomic analysis of the RT-qPCR-positive wastewater samples based on next-generation sequencing of PCR amplicons with the Illumina MiSeq® system confirmed the presence of the SARS-CoV-2 genome and identified variants.

Our WBE results from the village clearly indicated that a significant portion of asymptomatic carriers would not be identified through mass screening of travelers at airports by means of rapid antigen tests, unless additional strategies are employed, as pointed out by Torres.^4^ It is interesting to note here that the applicability of WBE to aircrafts of international flights has already been demonstrated,^5^ which can be an efficient tool to identify the presence of asymptomatic carriers and associated variants among air travel immigrants from a particular origin.

In conclusion, successful WBE implementation in the village clearly demonstrates its usefulness as a tool to control infections at other mass gatherings, such as future Olympic and Paralympic Games. We have demonstrated that WBE provides information beneficial for optimizing clinical testing schemes (e.g. prioritizing and determining the need for exhaustive tests) and for revealing the prevalence of known and unknown variants among asymptomatic patients, provided that proper sampling and analysis methods are employed.

## Authors’ contributions

Masaaki Kitajima: conceptualization, interpretation, writing-original draft; Michio Murakami: conceptualization, interpretation, writing-review and editing; Ryo Iwamoto: conceptualization, writing-review and editing; Hiroyuki Katayama: conceptualization, writing-review and editing; Seiya Imoto: conceptualization, interpretation, writing-review and editing.

## Authors’ statement

All authors have read and approved the published version of the manuscript.
